# Mesoporous graphene adsorbents for the removal of toluene and xylene at various concentrations and its reusability

**DOI:** 10.1038/s41598-019-47100-z

**Published:** 2019-07-29

**Authors:** Sun Taek Lim, Ji Hoon Kim, Chang Yeon Lee, Sangmo Koo, Dong-Wook Jerng, Somchai Wongwises, Ho Seon Ahn

**Affiliations:** 1 Department of Mechanical Engineering, Incheon National University, Incheon, Republic of Korea; 2 Department of Energy and Chemical Engineering, Incheon National University, Incheon, Republic of Korea; 3 School of Energy System Engineering, Chung-Ang University, Seoul, Republic of Korea; 4 Department of Mechanical Engineering, King Mongkut’s University of Technology Thonburi, Bangkok, Thailand

**Keywords:** Materials science, Chemical synthesis

## Abstract

As novel technologies have been developed, emissions of gases of volatile organic compounds (VOCs) have increased. These affect human health and are destructive to the environment, contributing to global warming. Hence, regulations on the use of volatile organic compounds have been strengthened. Therefore, powerful adsorbents are required for volatile organic compounds gases. In this study, we used graphene powder with a mesoporous structure to adsorb aromatic compounds such as toluene and xylene at various concentrations (30, 50, 100 ppm). The configuration and chemical composition of the adsorbents were characterized using scanning electron microscopy (SEM), N_2_ adsorption-desorption isotherm measurements, and X-ray photoelectron spectroscopy (XPS). The adsorption test was carried out using a polypropylene filter, which contained the adsorbents (0.25 g), with analysis performed using a gas detector. Compared to graphite oxide (GO) powder, the specific surface area of thermally expanded graphene powder (TEGP800) increased significantly, to 542 m^2^ g^−1^, and its chemical properties transformed from polar to non-polar. Thermally expanded graphene powder exhibits high adsorption efficiency for toluene (92.7–98.3%) and xylene (96.7–98%) and its reusability is remarkable, being at least 91%.

## Introduction

Volatile organic compounds (VOCs) cause photochemical reactions and various diseases, and have been designated as harmful to humans and the environment. The causes of VOC emissions can be classified as anthropogenic, such as the use of fossil fuels^1,2^, painting, transportation, architecture, petroleum products, polymer syntheses and printing ink^3,4^ and natural, including biological emissions from land or the ocean. In particular, as VOC emissions occur during daily activities, such as driving, cooking, smoking and cosmetics, they can cause serious human diseases, such as cancer, as well as headache and nausea; they can also induce unconsciousness. Thus, careful treatment is required, even in indoor environments^5,6^. For these reasons, efficient VOC removal methods and the development of suitable materials has become an important issue.

Many effective VOC removal methods, such as condensation, membrane separation, incineration, plasma catalysis, absorption, and adsorption have been developed in response to the tightening of worldwide environmental regulations, including the Kyoto protocol and the Paris agreement^7,8^. Unlike methods based on incineration or plasma catalysis, which emit toxic materials, adsorption is inexpensive, highly reusable and eco-friendly, and shows high performance. Hence, it is one of the most promising methods for removing VOCs. Many types of adsorbents with porous structures and large specific surface areas (SSAs), such as activated carbon (AC)^9–14^, biochar^15^, zeolite^16^, silicas^17^, AC fiber^18–22^, carbon nanotubes^23,24^ and metal–organic frameworks^25^ have been investigated to enhance VOC adsorption performance and clarify the adsorption mechanisms. Furthermore, to increase the number of reaction sites for VOCs, some chemical treatments, such as KOH activation^26,27^ and impregnation of metal oxide^28,29^ have been investigated. Li *et al*.^29^ carried out an adsorption experiment using toluene, acetone and 1,2-dichloroethane with three commercial AC samples, and discussed the relationship between adsorption capacity and pore volume of AC. Kwang *et al*.^11^ used granular AC (0.002 kg) to analyze the breakthrough curves of VOCs under atmosphere pressure. Baur *et al*.^28^ used modified ACFs with metal oxide to enhance the removal capacity of acetaldehyde. In terms of removing acetaldehyde, the performance of the modified ACFs improved by up to 20 wt.%. Kim *et al*.^27^ improved the adsorption performance for toluene from 80 to 98% using KOH activation, which can create more micropores and increase the SSA. However, there are several problems with the methods used to manufacture and modify adsorbents, such as high temperature requirements for manufacturing, incompatibility, low synthesis efficiency and high costs.

Many factors that can be adjusted to enhance physical adsorption or chemisorption performance, such as the SSA, pore size, pore distribution, molecular structure, polarity of adsorbent, boiling point, humidity and functional groups on the surface of the adsorbent. In particular, the SSA, pore size and functional groups on the surfaces of adsorbents are considered key factors affecting adsorption mechanisms, thus determining the VOC adsorption performance^30–33^. Large SSAs are important for the adsorption of VOCs, because they increase the number of adsorption sites. Hence, some researchers have focused on increasing the SSA. Chiang *et al*.^9^ evaluated three ACs with various SSAs, of 1,472 m^2^ g^−1^, 1,027 m^2^ g^−1^ and 975 m^2^ g^−1^, to investigate the variation in VOC adsorption performance with respect to the SSA. Fen-Yun Yi *et al*.^22^ increased the SSA of AC fibers using CuSO_4_ and demonstrated increased adsorption capacity for benzene, toluene, methanol and ethanol with modified ACF, which has a larger surface area. Lin Li *et al*.^12^ used chemical treatment to increase the surface area, which affects the adsorption capacity for non-polar VOCs on granular Acs, and observed a remarkable increase in adsorption capability. However, several researchers have reported that the highest SSA does not always lead to the best VOC adsorption performance. Gil *et al*.^32^ carried out a toluene removal test using various AC adsorbents, which were obtained by chemical activation with KOH, NaOH and K_2_CO_3_. They reported that the AC sample, which had a specific area of 798 m^2^ g^−1^, exhibited better toluene adsorption performance than another AC sample, which had a larger specific area of 1,169 m^2^ g^−1^. This indicates that adsorption performance is influenced by many related factors, and that these factors include not only the SSA, but other physical or chemical properties, including the pore size, polarity and functional groups present on the surfaces of adsorbents.

The pore size distribution also affects the adsorption performance of VOCs. The pore size can be classified into three categories according to diameter, i.e., micropores (pore diameter <2 nm), meso-pores (2 nm < pore diameter <50 nm) and macropores (pore diameter ≥50 nm). If the pore size is too big or small compared to the VOC gas molecules, the adsorption performance is poor. Hence, the relationship between pore size and adsorption capacity has been investigated by many authors using various methods. Yu-Chun Chiang *et al*.^9^ investigated the molar adsorption of VOCs such as benzene, chloroform, carbon tetrachloride and dichloromethane using AC with various mean pore sizes. M.A. *et al*.^33^ measured adsorption capability with respect to the pore size distribution and surface oxygen groups of AC. They reported that the porosity is a more important factor for determining VOC adsorption performance at low concentrations. Kun *et al*.^34^ conducted an adsorption test using MIL-101, which has a constant pore size, for various VOC gases with similar structures but different molecular sizes, such as ethylbenzene, p-xylene, o-xylene and m-xylene. According to their results, neither o-xylene nor m-xylene could be adsorbed at the surface of MIL-101 because its pores are too small. Hence, the appropriate pore size of the adsorbent varies with the target material.

In addition to the surface chemical functional groups of the adsorbents, the adsorbate has a profound influence on adsorption ability. In particular, almost all researchers have used engineered carbonaceous adsorbents with specific numbers of oxygen groups because this controls the acidity, which affects the interaction of polar or non-polar VOCs with the surfaces of adsorbents. Kim *et al*.^10^ fabricated modified AC using H_3_PO_4_ to increase the number of oxygen groups and enhanced the adsorption capability of VOCs such as methanol, ethanol and i-propanol. Similarly, acid treated biochar exhibited a better adsorption capacity for benzene and carbon tetrachloride than basic biochar^35^. Chemical activation, electrochemical treatment and microwave methods are commonly used to control the surface functional groups of carbonaceous adsorbents^27,36^. However, some researchers insist that the chemical functional groups at the adsorbent surface are not the key factor determining adsorption capacity. Tsai *et al*.^37^ investigated the adsorption capability of AC, which has a different polarity to chloroform. The adsorption capacity of the hydrophilic AC was approximately 373 mg/g, whereas it was 235 mg/g in the case of hydrophobic AC. Moreover, Gil *et al*.^32^ used an activated carbon which was treated by chemical materials as adsorbents for evaluating the capacity for toluene adsorption. The author insist that the adsorption capacities were affected by both textural properties and chemical functional groups.

Graphene, which consists of hexagonally arranged sp2-hybridized carbon atoms, has a remarkable theoretical SSA of 2,630 m^2^ g^−1^ ^38^. This can easily be altered by chemical reactions that modify the surface composition, such as the number of oxygen functional groups, and can be manufactured using tape^39^, chemical vapor deposition^36^, hydrothermal processes^40^, etc. Hence, graphene is considered to be a promising adsorbent material due to its large specific area, various production techniques and ease of modification. So, graphene had been used for adsorbent with various chemical modifications^41,42^. Furthermore, some researchers have reported good adsorption of VOCs with benzene rings, such as benzene, toluene and xylene. The adsorption mechanism has been explained in terms of π-π electron donor-acceptor (EDA) reactions^43^. However, many studies on graphene have focused on the removal of VOCs from water, and there have been few studies on techniques for removing them from air, where they are easily introduced due to pollution.

Toluene and xylene are among the toxic materials emitted from factories and petrochemical products and are subject to worldwide emissions regulations. Thus, research and development of adsorbent materials that are applicable to real-life scenarios is a serious issue. Hence, in this study, we used bulk graphene powders with mesoporous structures as adsorbents. The graphene bulk powders were produced by the thermal expansion method (resulting in thermally expanded graphene powder, TEGP) and characterized by scanning electron microscopy (SEM), X-ray photoelectron spectroscopy (XPS) and N_2_ isotherms. Without using any bonding materials, 0.25 g of TEGP was sealed in a circular air filter with no VOC adsorption properties. We assessed the adsorption whilst varying the VOC concentration (30, 50, 100 ppm) and measured the total adsorbed volume capacity. Finally, we verified the economic feasibility of TEGP by carrying out a reusability test.

## Experimental Section

### Preparation of thermally expanded graphene powder

GO powder was prepared using a modified Hummers’ method^44^. To oxidize the graphite powder (2 g, flake type, ~325 mesh; Alfa Aesar), chemical materials such as sodium nitrate (1 g; Sigma-Aldrich), sulfuric acid (69 ml; Daejung), potassium permanganate (6 g; Sigma Aldrich), hydrogen peroxide (3 ml; Daejung) and deionized (DI) water were used, followed by a freeze-drying process. We treated GO powder with thermal energy to form a porous structure. At the initial condition of room temperature (25 °C), an alumina boat containing GO powder (0.5 g, brown color) was placed in a tube furnace. The inside of the tube furnace was composed of an argon environment using vacuum pump and argon bombe. During manufacture process the argon gas was flowed with 0.5 L/min. Until reach to manufacturing temperature as 200, 500 and 800 °C, the heat rate was kept constant at 5 °C/min. After the conditions of tube furnace reached the target temperature, the value was maintained for 30 minutes and then cooled. Finally, we obtained low density thermally reduced oxide graphene powder (black) with a maximal volume. This black powder was named as TEGP. Depending on the applied temperature, the concentration of oxygen molecules varied due to the elimination of functional groups, such as carboxyl and carbonyl groups^45^.

### Characterization

We observed the morphology of the TEGP using field emission scanning electron microscopy (FE-SEM; JEOL-7800F). The acceleration voltage was up to 30 kV, at which the resolution was guaranteed to be 1.2 nm. The magnification of the SEM images was selected as ×2,000 or ×20,000. The nitrogen adsorption-desorption isotherm measurement (Autosorb-iQ; Quantachrome Instruments) was carried out at 77 K. All powders were pre-activated at 120 °C under vacuum conditions to remove water from the powder; then, VOC adsorption-desorption measurements (BELSOP-man; MicrotracBEL Corp.) were carried out using toluene and xylene so that we could investigate the total adsorbed volume capacity of TEGP. We analyzed the chemical composition of TEGP using XPS (PHI 5000 VersaProbe-II; Phi) so that we could compare the concentrations of oxygen and carbon.

### VOC gas adsorption test

**Figure 1:**
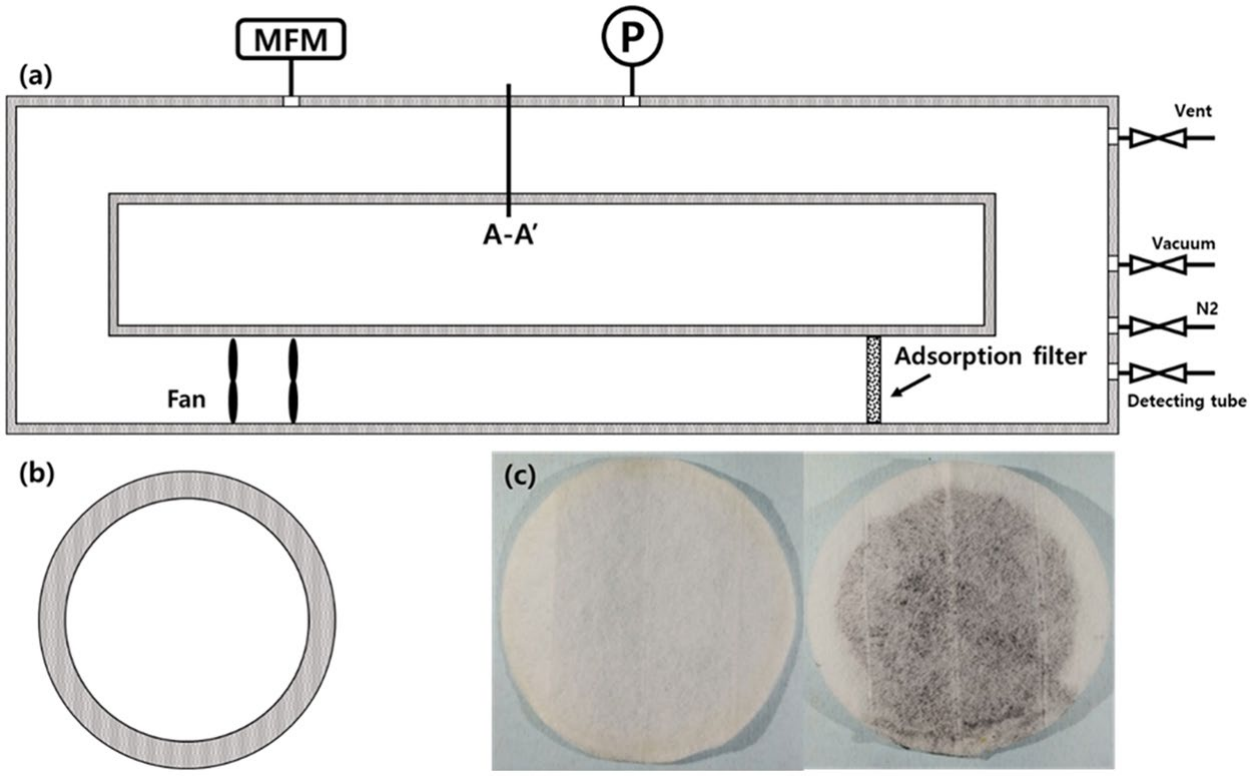
(**a**) Schematic of apparatus and components (**b**) The cross section of A-A’ section (**c**) picture of PP-filter (left) and adsorption filter containing thermally expanded graphene powder, TEGP (right).

The loop for VOC gas adsorption consisted of an acrylic chamber, electrical fan for circulating gas, test section, pressure gage and flow meter. The volume of the acrylic chamber, which had a circular cross section, was 5,570 cc and all tubes had a diameter of 60 mm. The electrical fan, which could produce flow rates of 2.23 m^3^ min^−1^ allowed circulation of VOC gas and accelerated the reaction between the VOC gas and the TEGP. A polypropylene (PP) air filter, which had a diameter of 70 mm and no adsorption capability, was used to enclose the TEGP. The pressure at the acrylic chamber was measured using a pressure gauge and maintained at 0.1 MPa (gauge pressure) during the adsorption test. We checked for gas leaks in the pressurized acrylic chamber by immersing it in a water pool. We controlled the gas supply and venting using valves with 1/4 inch holes, and their concentrations were monitored using a gas detector (GASTEC). The flow rate of gas was monitored in real-time by a flow meter. All gaps or contact surfaces were sealed using O-rings and silicon sealant (SYLGARD 184; Dow Corning). Figure 1(a,b) show a schematic of the overall acrylic chamber and cross section of the tube, respectively. The width of the apparatus was 1,040 mm and its depth was 190 mm. For precise measurement of the mass flow rate, we selected the size of the apparatus based on the guidelines of the measurement devices.

We designed the experimental cases by considering the type of VOC gas (toluene, xylene) and its concentration (30, 50, 100 ppm). We compared the adsorption performance of the different concentrations by wrapping the GO powder and TEGP with PP filters and carrying out adsorption tests. Each of the experimental cases are detailed in Table 1. We prepared the adsorption filter by enclosing the TEGP powders with basic PP filters. We overlapped two pieces of PP filter with diameters of 70 mm and bonded them together. After 0.25 g of TEGP had been placed in the PP pocket, we bonded the pocket to ensure that it was completely sealed. Although gas passes through the adsorption filter, we never observed TEGP leakage from the filter, thus confirming that the filter prevents the loss of adsorbents. Hence, this simple method of enclosing the material in a basic filter is an effective strategy for preparing adsorption filters at industrial or lab scales. Figure 1(c) shows a reference filter, which does not contain TEGP, and the adsorption filter with TEGP powder fabricated at 800 °C.

**Table 1:** Experimental cases.

Material	Manufacture method	Annealing temperature [°C]	Name	VOC gas	Concentration [ppm]
Graphite oxide	Oxidation	—	GO powder	Toluene, xylene	30, 50, 100
TEGP	Thermally annealing	200	TEGP200	Toluene, xylene	30, 50, 100
TEGP	Thermally annealing	500	TEGP500	Toluene, xylene	30, 50, 100
TEGP	Thermally annealing	800	TEGP800	Toluene, xylene	30, 50, 100

The VOC gas adsorption test was carried out as follows. The acrylic chamber containing the adsorption filter was filled with nitrogen gas and we then created a vacuum environment by removing the N_2_ gas using a vacuum pump. This process was repeated three times to guarantee a vacuum environment. After the acrylic chamber was filled with VOC gas, such as toluene or xylene, the adsorption tests were started. The adsorption performance was evaluated by gas detector and detecting tube (GASTEC) which indicates the amount of VOC gas mixed in the N_2_ gas. The detecting tube has scales which indicate the amount of VOC gas. The evaluation processes for adsorption performance as follow: link the detector tube at the front of gas detector and connect the tube in the opposite direction to the experiment apparatus. After that, if the grip which was located at end of the gas detector was pulled, the color of detecting material which was located inside of detecting tube will be changed up to the scale of the amount containing the VOC gas as brown color. We classified the brown color by image processing program with the same basis to determine the amounts of adsorbed gases. We verified the adsorption performance of the PP filter by repeating the adsorption test using a PP filter containing no carbon powder. The results of this test confirm that the adsorption performance was similar in all cases, except when we compared the case with GO powder to that of TEGP. After the end of the test, the remaining gas in the acrylic chamber was removed using a vacuum pump. We repeated the same measurement procedures, replacing the PP filter each time. All adsorption tests were carried out at a constant room temperature (25 °C) under zero humidity conditions.

### Reusability

After completing the adsorption test, we fixed the TEGP filter to the aluminum desorption apparatus to evaluate its reusability (the desorption apparatus is shown in Fig. S1). To eliminate VOC gases, which were adsorbed by the TEGP, we used a fitting tube with a diameter of 6 mm to connect the nitrogen supply line and vent line to each end of the desorption apparatus, then placed it in a convection oven to achieve a constant temperature of 150 °C. The nitrogen gas flowed steadily through the TEGP filter for 30 min. After the desorption process, the TEGP filters were installed in adsorption apparatus and the adsorption test was repeated, as described above. We then validated the re-adsorption performance using a gas detector. Finally, we compared the results to those obtained prior to the desorption test.

## Results and Discussion

### TEGP characterization

**Figure 2:**
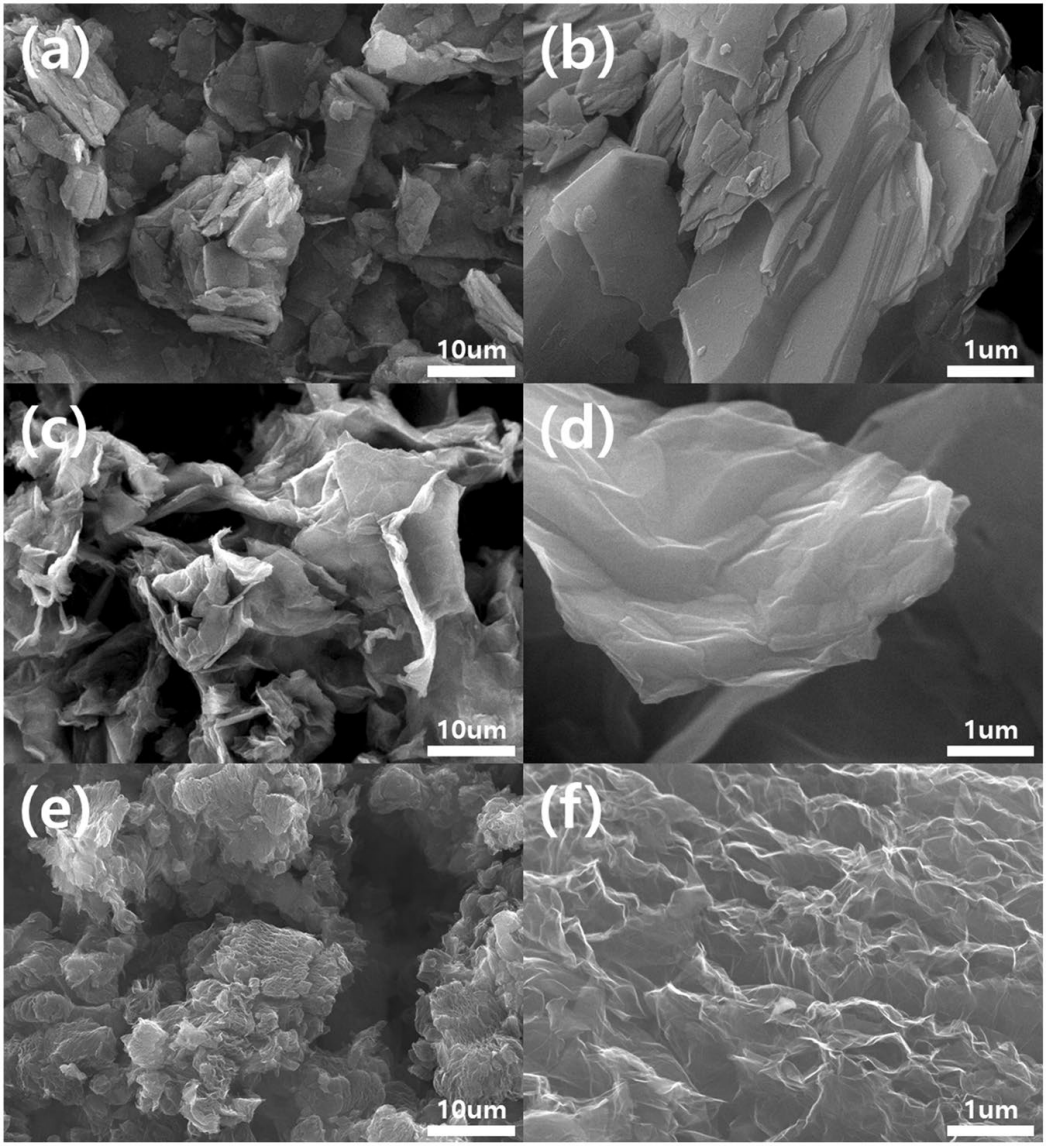
Scanning electron microscopy (SEM) image of (**a**,**b**) graphite powder, (**c**,**d**) graphite oxide (GO), and (**e**,**f**) TEGP800.

**Figure 3:**
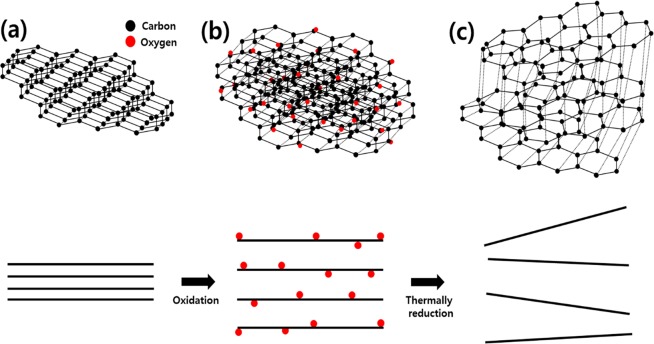
Schematic of the thermal expansion mechanism; (**a**) graphite powder, (**b**) GO powder, and (**c**) TEGP.

Figure 2 shows the morphologies of the adsorption materials [graphite, (a), (b); graphite oxide, (c), (d); TEGP800, (e), (f)] for VOC gas, with various magnifications [(a), (c), (e), ×2,000; (b), (d), (f), ×20,000]. As shown in Fig. 2(a–d), a porous morphology was not observed in the case of the graphite and GO powder, which was composed of multiple layer stacked graphene powder. However, due to the oxidation and rinsing process, many more wrinkles were generated on the surface of GO powder compared to the graphite powder. The SEM image of TEGP800 (Fig. 2(e,f)), which contains expanded gaps between the multi-layer graphene sheets, shows that the porous structure arose due to the thermal energy treatment, also TEGP200 and TEGP500 show similar morphologies. The pores in the TEGP were generated by the emission of oxygen functional groups, such as carbonyl groups, that were present on the surface of the graphene layer. This method, based on thermal energy, can be applied to facilitate instant redox processes, and induce the generation of micro-meso porous cavities and oxygen groups, which are related to polarity; also, it can be easily controlled by adjusting the temperature^45^. The interlayer spacing between the graphene sheets was increased by the adhesion of oxygen molecules during the oxidation treatment. Furthermore, the SSA at the adsorption sites was maximized using a thermal reduction process at 800 °C, thus removing the intercalated oxygen atoms. Figure 3 shows a schematic of the thermal expansion mechanism.

**Figure 4:**
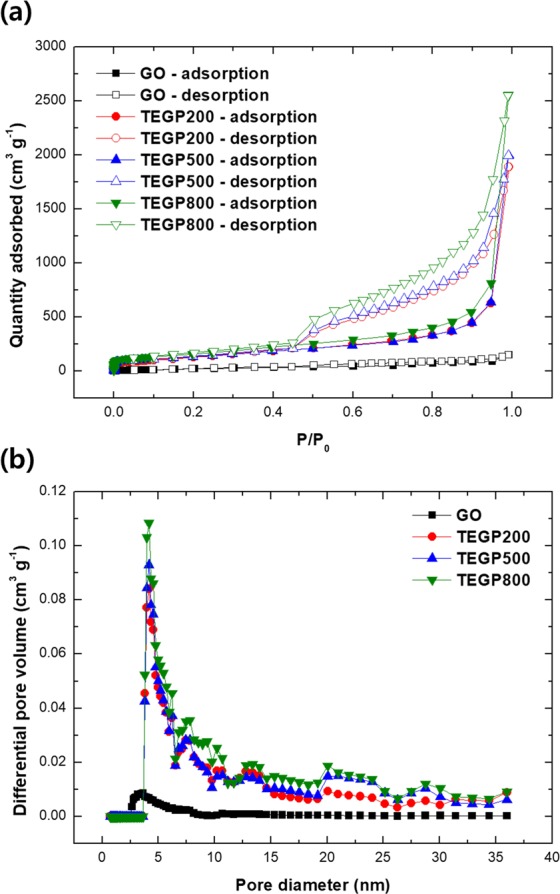
(**a**) Nitrogen adsorption-desorption graph and (**b**) pore distribution of adsorbents (Black: GO powder, Red: TEGP200, Blue: TEGP500, Green: TEGP800).

Figure 4(a,b) show the results of the nitrogen adsorption-desorption isotherm measurements and the pore distribution, respectively (graphite oxide, black line; TEGP200, red line; TEGP500, blue line; TEGP800, green line); filled and black symbols in (a) indicate adsorption and desorption, respectively. The SSA of GO, TEGP200, TEGP500, TEGP800 were measured as 65, 414, 440, 542 m^2^ g^−1^, respectively. The SSA of the TEGP were increased according to treating temperature and were maximized at 800 °C. The specific surface area of TEGP800 was 8–9 times larger than that of the GO powder. As shown in Fig. 4(a), the relative pressure region between 0.0–0.1 indicates powerful adsorption ability, which was caused by the surface of the adsorbent. The higher relative pressure indicates that more bulk gas was adsorbed by the limited volume of adsorbent. The TEGP generally adsorbed a large quantity of nitrogen due to its larger SSA and volume for gas adsorption. The adsorption-desorption graph for all case of TEGP shows type H3 isotherms, which means that the adsorbents have slit-like pores^46^; this pore shape is shown in Fig. 2(e,f). As shown in Fig. 4(b), the pore distribution graph of the GO power indicates a lower porosity, whereas the graph for all case of TEGP indicates the generation of mesopores of various sizes; in particularly, the quantity of mesopores with diameters from 4 to 5 nm increased remarkably, and pores with diameters between 5 to 25 nm were well-developed. This indicates that the application of thermal energy to the GO powder affected the form of the porous structure and shows that a mostly mesoporous structure can be fabricated quickly and easily by applying the thermal expansion method to the bulk material.

**Figure 5:**
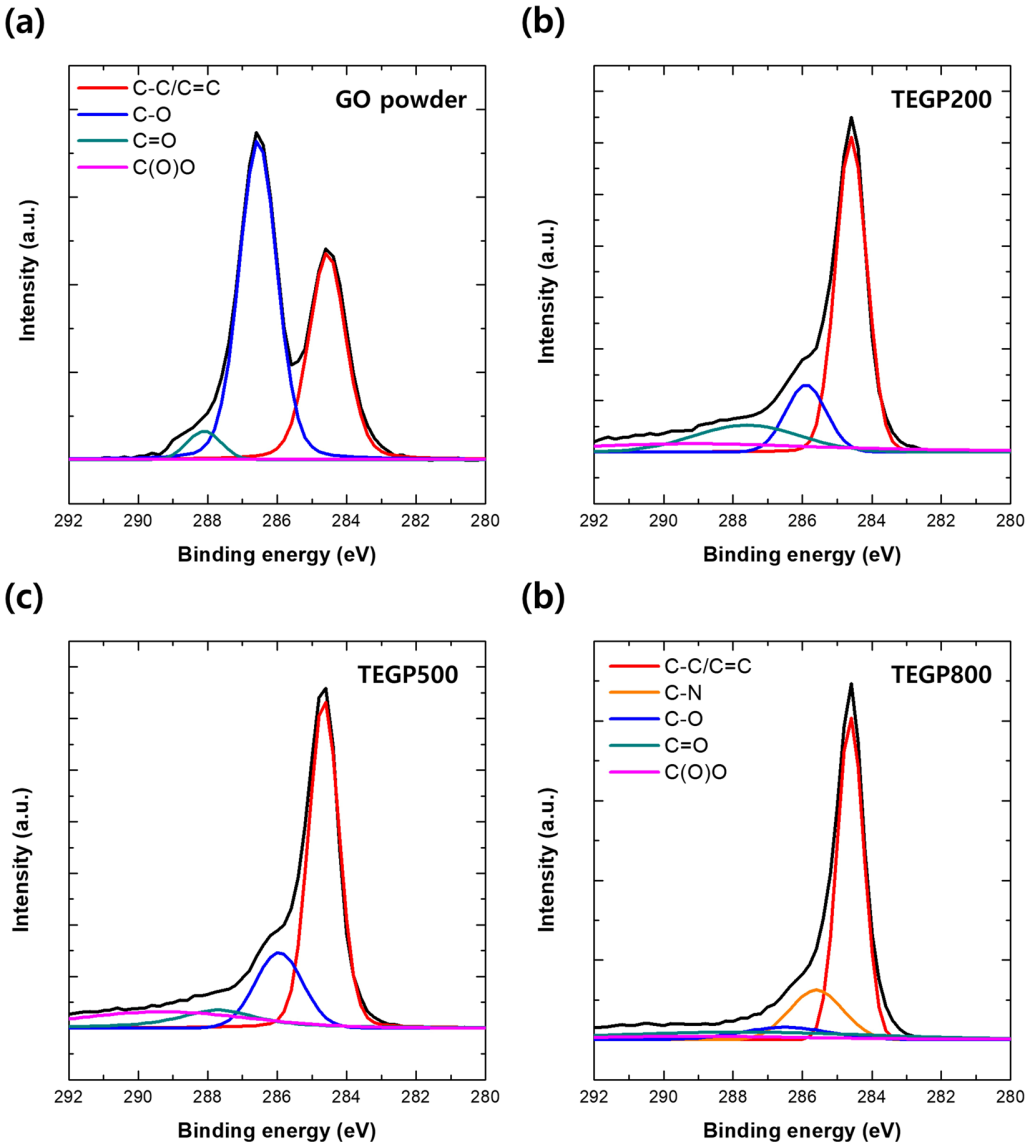
XPS data of C1s peak separation of (**a**) GO powder, (**b**) TEGP200, (**c**) TEGP500, (**d**) TEGP800.

Figure S2 shows the XPS results, namely the intensity (a.u.) against the binding energy (eV), to show the variation in O1s and C1s peaks with respect to the TEGP processing temperature (black: GO powder, red: TEGP200, blue: TEGP500, green: TEGP800); we refer to the TEGP manufactured at 200 °C as TEGP200, *etc*. The results show that the intensity of the O1s peak decreased with increasing temperature, unlike the C1s ratio, which increased with respect to temperature. This indicates that functional groups containing oxygen, such as hydroxyl- and carboxyl-groups, were removed effectively from the surface of the GO powder. The variation in oxygen ratio with respect to temperature demonstrates that the removal of the functional groups, which contain oxygen atoms, depends on the temperature. The C1s peaks of GO, TEGP200, TEGP500, TEGP800 were separated, as shown in Fig. 5(a–d), respectively. These show the change in chemical composition in detail. Comparing the GO powder with every TEGP case, the intensity of the C–O and C=O bonds decreased remarkably. We calculated the amount of oxygen functional groups which were remained on the adsorbent surface. The C/O ratio of GO, TEGP200, TEGP500 and TEGP800 were calculated as 0.6, 1.3, 1.3 and 2.1 and the percentages of oxygen-functional groups were calculated as 62.7, 44.1, 44.0 and 26.7%, respectively. As the data shows, oxygen functional groups which were existed on the surface were removed greatly but not completely. This indicates that the thermal energy-based reduction method affected the sp2- bonding of the graphene plane. In our analysis of the TEGP with respect to the annealing temperature, the amount of the oxygen-functional groups decreased according to the manufacturing temperature such as 200, 500 and 800 °C. This indicates that the annealing conditions can be controlled to adjust the ratio of oxygen at the graphene plane, thus affecting the polarity.

### Adsorption performance of VOC using adsorbents

We carried out our VOC gas adsorption experiment using GO, TEGP200, TEGP500 and TEGP800 with toluene and xylene gas in an acrylic chamber at various concentrations (30, 50, 100 ppm). The two overlapping pieces of bare PP filter were used as an enclosing material to prevent the adsorbents from escaping the PP filter. Prior to the adsorption experiments, we confirmed that the bare PP filter adsorbed no VOCs at a concentration of 100 ppm.

**Figure 6:**
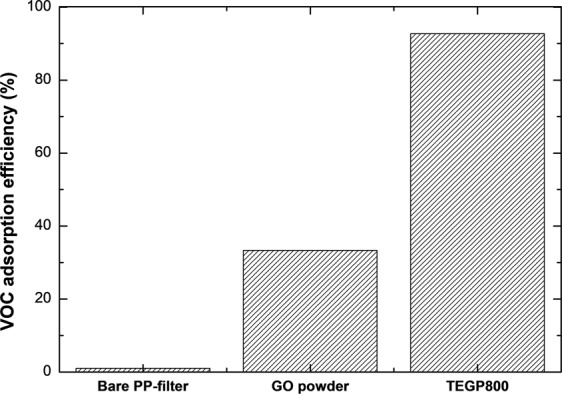
VOC adsorption efficiency of bare PP-filter, GO powder and TEGP800 for toluene.

Figures S3 and S4 show the adsorption efficiency of TEGP 200, 500 and 800 according to VOC gas type. Although the SSA and containing amounts of oxygen-functional groups are different at each TEGP, the adsorption performances are similar. This is because the pore distribution of each TEGP and the dominant pore size are similar^33,47^. So, we compared GO power and TEGP800 as representative. Figure 6 shows the adsorption performance (%) of the adsorption filter, with various adsorbents [(a), bare PP-filter; (b), GO powder; (c), TEGP800] for 100 ppm of toluene gas. Our experimental results show that the bare PP-filter adsorbed almost no toluene gas. This confirms that the bare PP-filter is suitable for enclosing adsorbents, because it does not affect the adsorption performance. The TEGP800 exhibited outstanding toluene adsorption performance (93%), which was also better than that of GO powder (33.3%). This confirms that the differences between physical properties of the GO powder and TEGP800, such as the larger SSA, volume of adsorbent, presence of chemical functional groups at the surface of the adsorbent and presence or absence of pore, have a remarkable effect on the adsorption performance.

**Figure 7:**
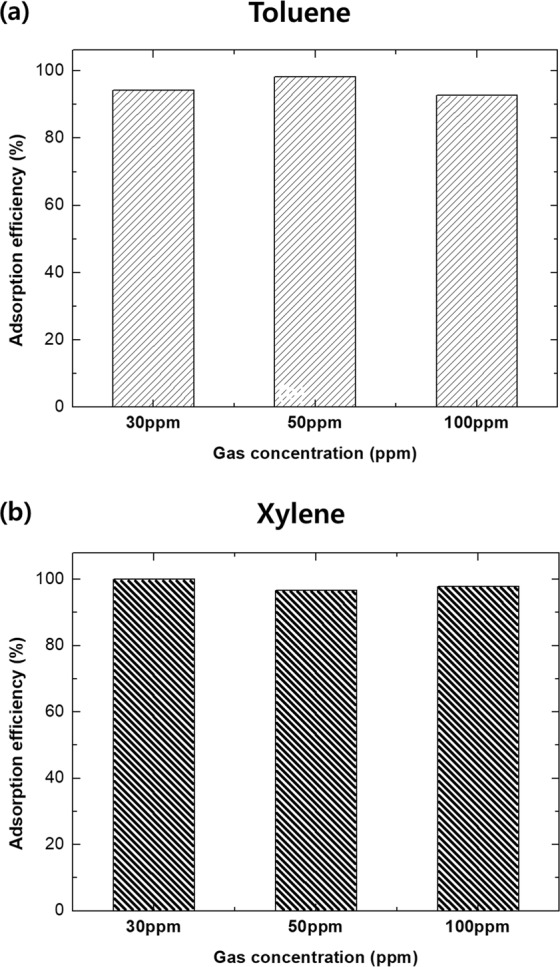
Adsorption efficiency of TEGP800 with respect to the gas concentration of (**a**) toluene and (**b**) xylene.

Figure 7(a,b) show the toluene and xylene adsorption performance (%) of TEGP800, demonstrating the effect of gas concentration. According to our results, toluene and xylene adsorption did not vary significantly with the gas concentration, as shown in Figs S3 and S4. The adsorption efficiency peaked after 30 min in the case of both toluene and xylene. As a representative, the adsorption performance of TEGP800 at 30, 50, 100 ppm was, respectively, 94.3, 98.3, 92.7% for toluene gas and 100, 96.7, 97.9%, for xylene gas. There were some differences in adsorption performance of toluene compared to the results shown in Fig. 6. The performance was significantly improved compared to that of GO powder.

**Figure 8:**
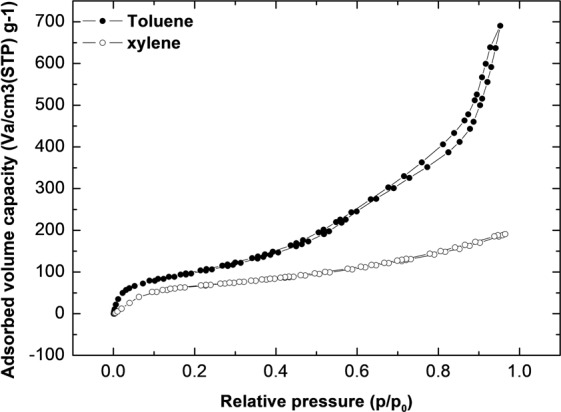
Total adsorbed capacity of TEGP800 for toluene (filled circle) and xylene (blanked circle).

Figure 8 [(a): toluene, (b): xylene] shows the VOC gas adsorption-desorption trend, which we used to verify the total adsorbed volume capacity of TEGP800. This shows that the total adsorbed volume capacity of TEGP800 was 691 and 191 cm^3^ g^−1^ for toluene and xylene, respectively. The VOC gases used in the adsorption-desorption tests have molecular structures with methyl groups. It is possible that the differences in adsorbed volume capacity mentioned are due to the volume of each VOC molecule. Hence, as the total TEGP adsorption volume is limited, it is reasonable to assume that larger molecule size results in smaller total adsorbed volume capacity, as shown in Fig. 8. This figure suggests that the density of toluene and xylene is 867 and 861 kg/m^3^, respectively; these values are reasonable.

### Reusability test

**Figure 9:**
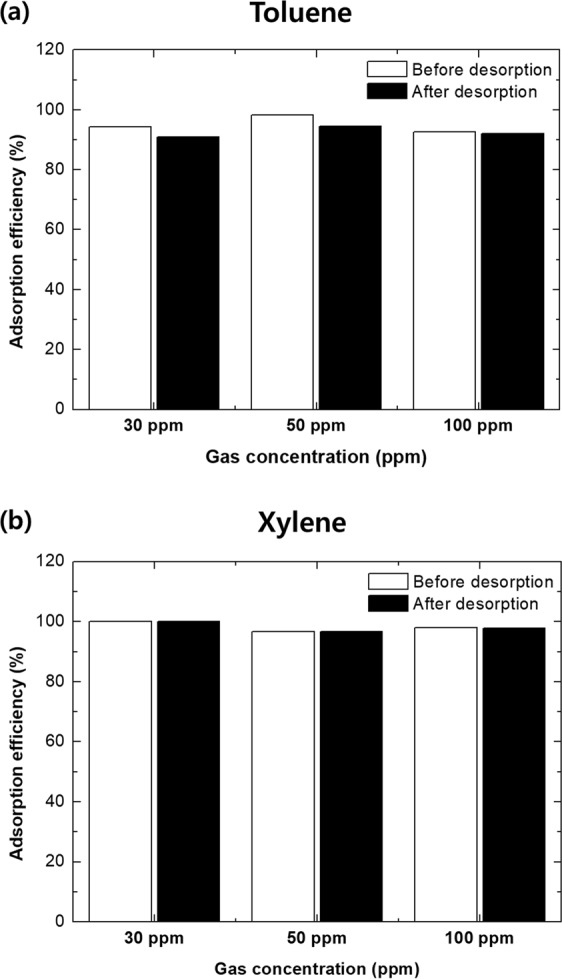
Comparison of adsorption efficiency of TEGP800 before versus after the desorption process for (**a**) toluene and (**b**) xylene.

When developing adsorption methods, it is necessary to consider reusability, which is one of the most important factors when evaluating intrinsic adsorbent performance. Regardless of the quality of the adsorption performance, if a material is not reusable, then the adsorbent is significantly less attractive from an economic and environmental point of view. We investigated the reuse potential of TEGP200, 500 and 800 by first placing the desorption apparatus in an convection oven to realize constant temperature conditions. The thermal energy made it easy to remove both the physically adsorbed VOC gas molecules and the chemically adsorbed gas molecules through processes such as the π-π interaction. Figures S5 and S6 show the re-adsorption efficiency of TEGP 200, 500 and 800 according to VOC gas type. Likewise, the adsorption efficiency, all of TEGP samples show similar re-adsorption efficiency. In the case of TEGP800 as a representative, it shows that the reused TEGP800 had superior toluene and xylene re-adsorption efficiency at all gas concentrations, of 91 and 97% or more, respectively, although there was a slight drop in efficiency prior to reuse. The re-adsorption efficiency also became saturated after 30 minutes and showed good adsorption efficiency. This indicates that the TEGP adsorption performance was recovered by the thermal energy-based desorption method. Figure 9(a,b) show a comparison of the adsorption efficiency of TEGP800 between toluene and xylene before and after the desorption process, respectively. In the case of the toluene gas, the adsorption efficiency decreased slightly, by approximately 0.7–3.8%, when the TEGP was reused. There was no decrease in adsorption efficiency after versus before the desorption process in the case of xylene gas; hence, the xylene adsorption efficiency of the TEGP800 was restored almost perfectly. Although we observed a slight decrease in the adsorption efficiency when we used TEGP samples, it exhibited outstanding toluene and xylene reusability properties, i.e., greater than 96%.

The adsorption performance of aromatic VOC gases with benzene rings proceeds by the EDA reaction, involving π electrons on the graphene surface, aromatic rings, and oxygen functional groups^31,45^. In this study, with toluene and xylene, which are non-polar, the adsorption performance of TEGP exhibited remarkably superior adsorption efficiency, from low concentrations (30 ppm) to high concentrations (100 ppm), compared to GO powder. This was due to the maximized SSA of the TEGP and the elimination of the oxygen functional groups and trend of pore size distribution. Eventually, although all of TEGP samples have a different specific surface area and amounts of oxygen-functional groups, the adsorption efficiency was showed similarly. This is because TEGP200, 500 and 800 have similar pore size that exists dominant^33,47^. Furthermore, the thermal expansion method, which we used to control the SSA and functional groups, provides an economical manufacturing method because it barely emits any harmful substances and is simple and easy to use. We achieved outstanding VOC adsorption efficiency with TEGP800, of approximately 93%, which became saturated after 30 minutes. The total adsorbed capacity was greater in the case of toluene (691 cm^3^ g^−1^) than in the case of xylene (191 cm^3^ g^−1^), indicating that toluene has advantages over xylene in terms of gas molecular structure and size. Finally, the reusability of the TEGP was remarkable, being greater than 96%. This indicates that this material had outstanding properties for applications as an adsorbent for VOCs.

## Conclusions

In summary, we manufactured TEGP, which is an appropriate material for the removal of VOC gas, by a chemical oxidation process and reduction using heat energy. The adsorption performance of the toluene and xylene was evaluated using a gas detector tube (GASTEC) under concentrations of less than 100 ppm. Furthermore, the economic potential of TEGP was evaluated in terms of its reusability. The findings of this study can be summarized as follows:

- The dominant pore size of the adsorbent is a key factor affecting the adsorption performance. We carried out reduction using heat energy to control the specific surface area of the adsorbent, oxygen-functional groups and pores. To analyze the controlled factors, we used the Brunauer—Emmett—Teller (BET) method and X-ray photoelectron spectroscopy (XPS). TEGP has been identified previously as having a porous structure and a significantly increased SSA based on SEM and BET data and the oxygen-functional groups were decreased from 62.7 to 26.7%. But the adsorption efficiency is not significant difference. This is because all of TEGP samples have similar dominant pore size with 4–5 nm.
- TEGP exhibited remarkable adsorption performance at each concentration of toluene and xylene gas tested (30, 50, 100 ppm). But, TEGP had a large difference in the total adsorbed volume capacity of toluene and xylene gas. This shows that the VOC gas adsorption performance is not only determined by the SSA.
- We investigated the total adsorbed volume capacity, which was 691 cm^3^ g^−1^ in the case of toluene gas and 191 cm^3^ g^−1^ in the case of xylene gas, using a BET method. These results are reasonable given that the molecular size of toluene is smaller than that of xylene, so more gas can be adsorbed at a given volume.
- We also investigated the reusability of TEGP by carrying out a desorption and re-adsorption test. To desorb the VOCs molecules effectively, we placed the desorption apparatus in a convection oven, which was maintained at a constant temperature of 150 °C. Having achieved a re-adsorption efficiency over 91%, we speculate that the TEGP filter clearly maintains its sorption function. Although we observed a slight decrease in adsorption efficiency, of approximately 0.7–3.8%, the adsorption upon reuse was greater than 96% in the case of both toluene and xylene. This indicates that TEGP has excellent reusability properties.

## Supplementary information

Supplementary materials
