# Supplementary material for: Mesoporous graphene adsorbents for the removal of toluene and xylene at various concentrations and its reusability
Source: Sci Rep. 2019 Jul 29;9:10922. doi: 10.1038/s41598-019-47100-z (PMC6662692; doi:10.1038/s41598-019-47100-z)
Supplement: Supplementary file 1 — Supplementary materials [file 41598_2019_47100_MOESM1_ESM.pdf]

Supplementary Information

Mesoporous graphene adsorbents for the removal of toluene and xylene at various concentrations and its reusability

Sun Taek Lim[1], Ji Hoon Kim[1], Chang Yeon Lee[2], Sangmo Koo[1], Dong-Wook Jerng[3], Somchai Wongwises[4], and Ho Seon Ahn[1*]

[1]Department of Mechanical Engineering, Incheon National University, Incheon, Republic of Korea

[2]Department of Energy and Chemical Engineering, Incheon National University, Incheon, Republic of Korea

[3]School of Energy System Engineering, Chung-Ang University, Seoul, Republic of Korea

[4]Department of Mechanical Engineering, King Mongkut's University of Technology Thonburi, Bangkok, Thailand

Desorption apparatus

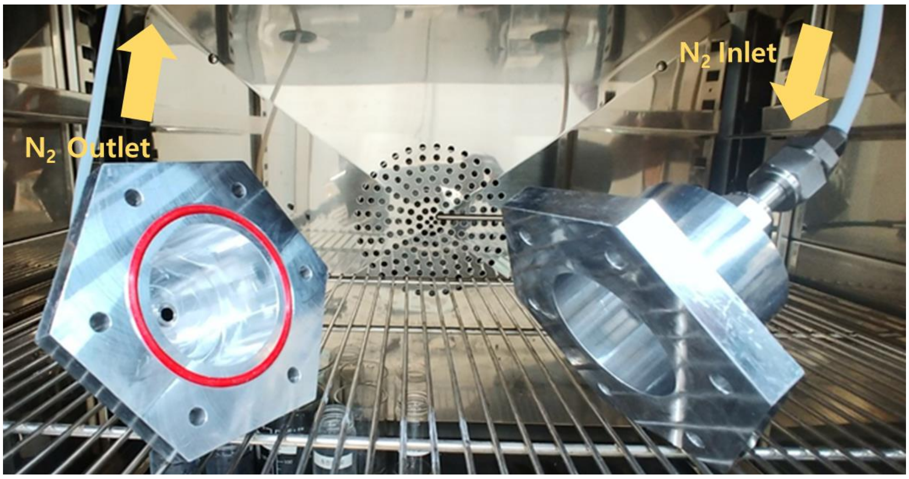

Figure S1. Desorption apparatus for reusability

The XPS spectra survey data

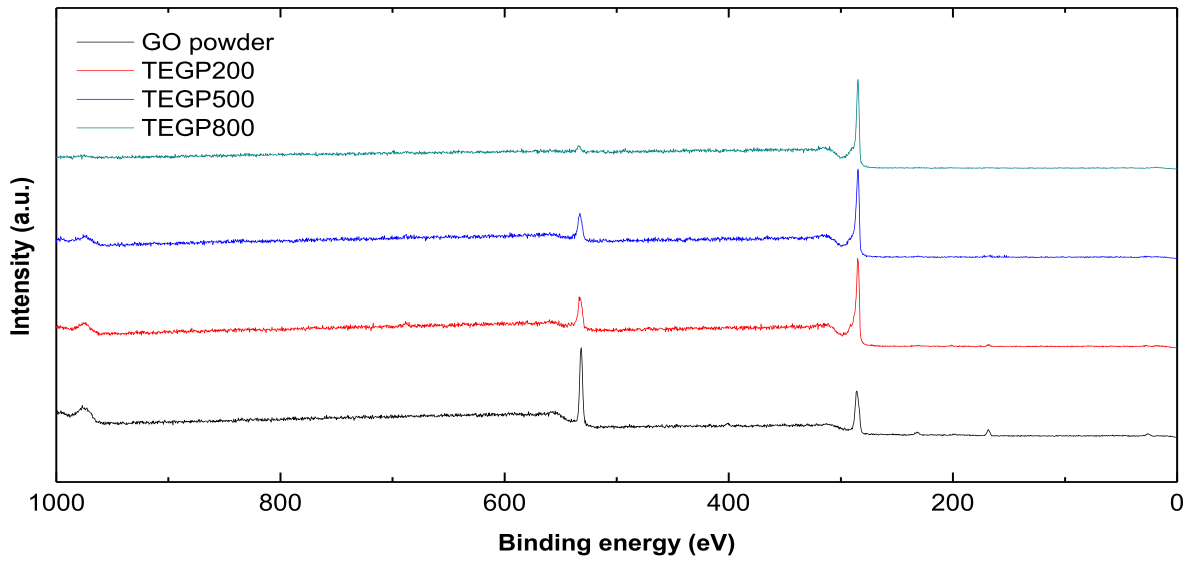

Figure S2. The XPS spectra survey data of intensity over binding energy for the peak of O1s and C1s according to fabricated temperature of TEGP (Black: GO power, Red: TEGP200, Blue: TEGP500 and Green: TEGP800).

Toluene adsorption efficiency versus time according to type of TEGP

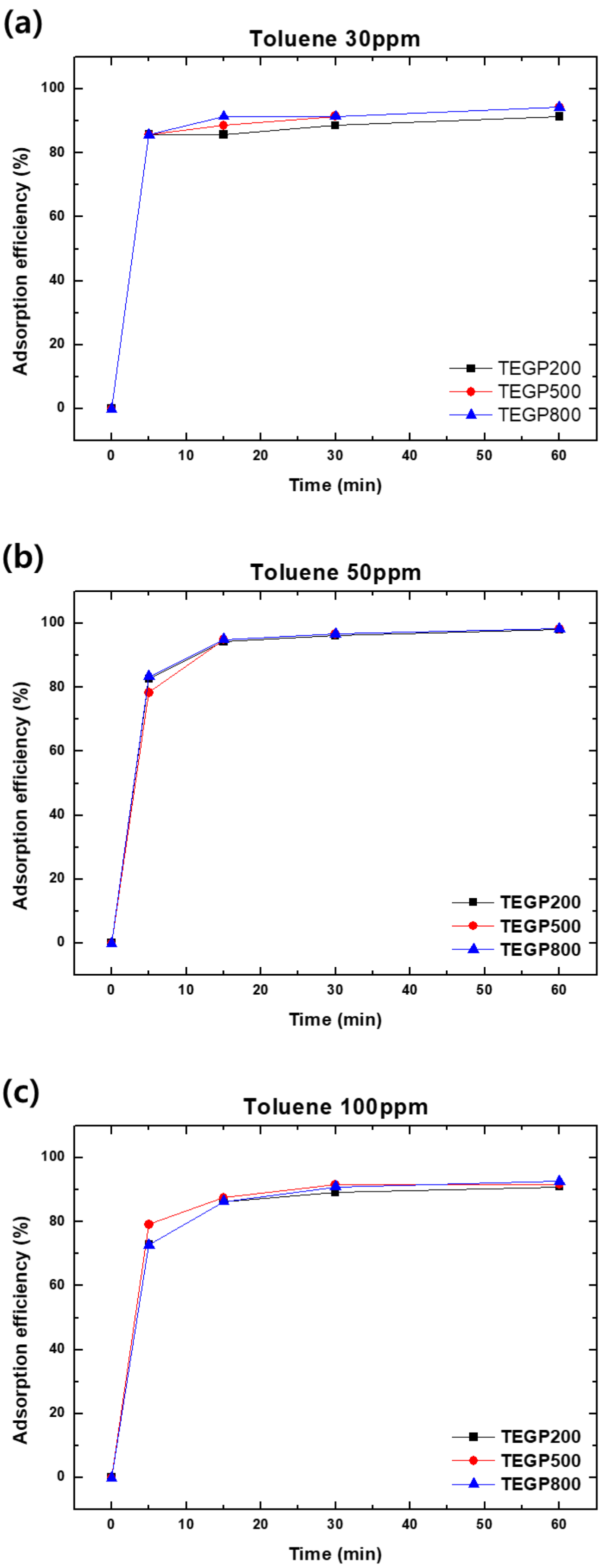

Figure S3. Toluene adsorption efficiency according to type of TEGP (a) 30ppm, (b) 50ppm and (c) 100ppm.

Xylene adsorption efficiency versus time according to type of TEGP

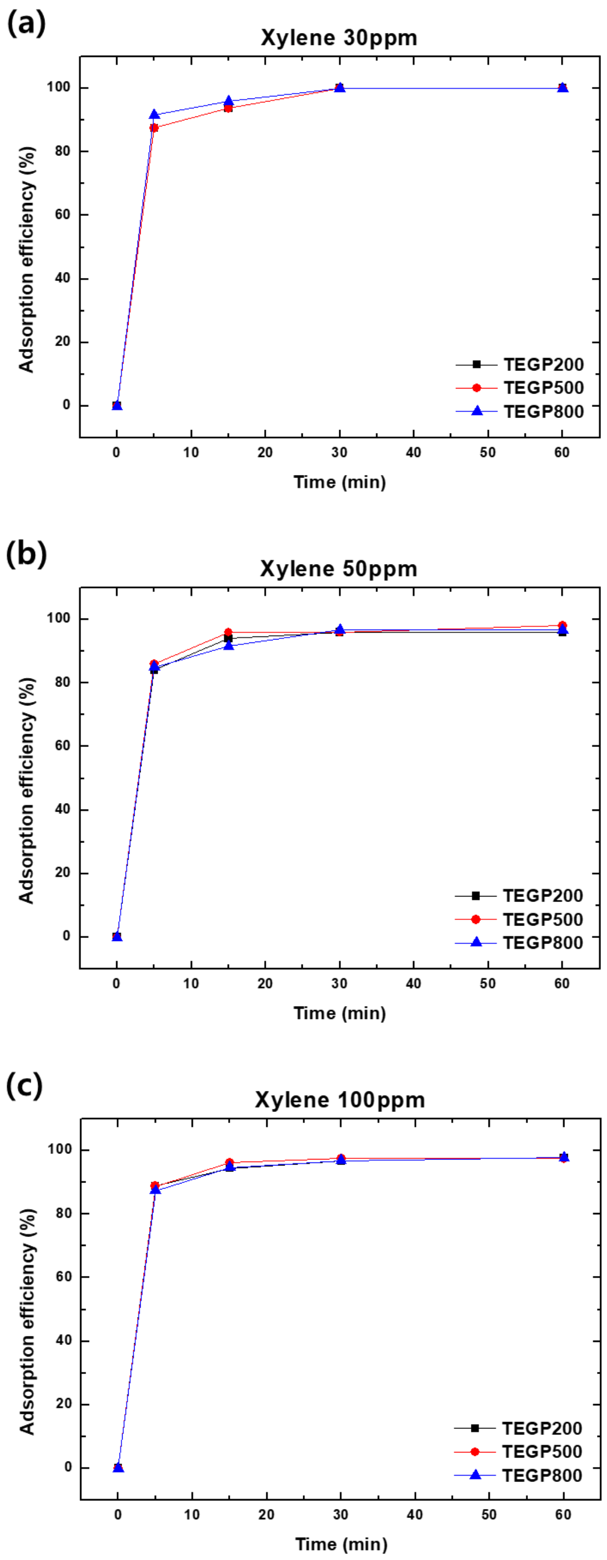

Figure S4. Xylene adsorption efficiency according to type of TEGP (a) 30ppm, (b) 50ppm and (c) 100ppm.

Toluene re-adsorption efficiency versus time

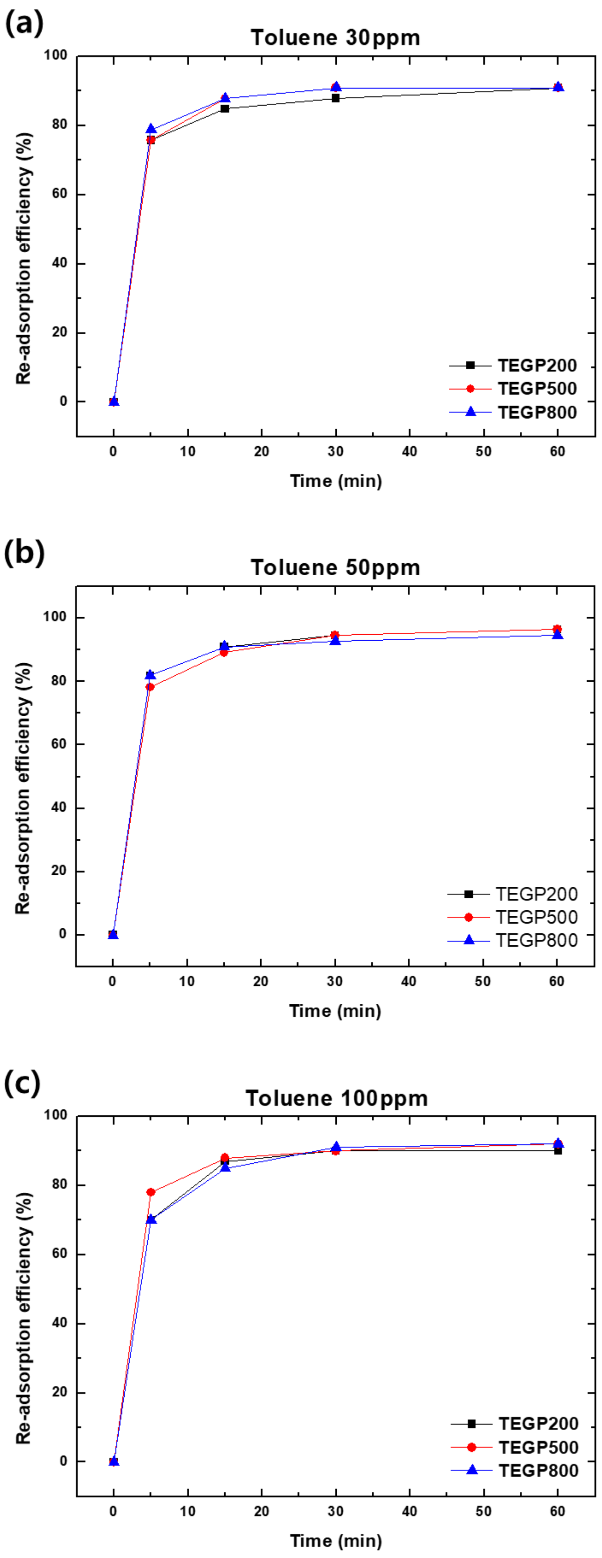

Figure S5. Toluene re-adsorption efficiency according to type of TEGP (a) 30ppm, (b) 50ppm and (c) 100ppm.

Xylene re-adsorption efficiency versus time

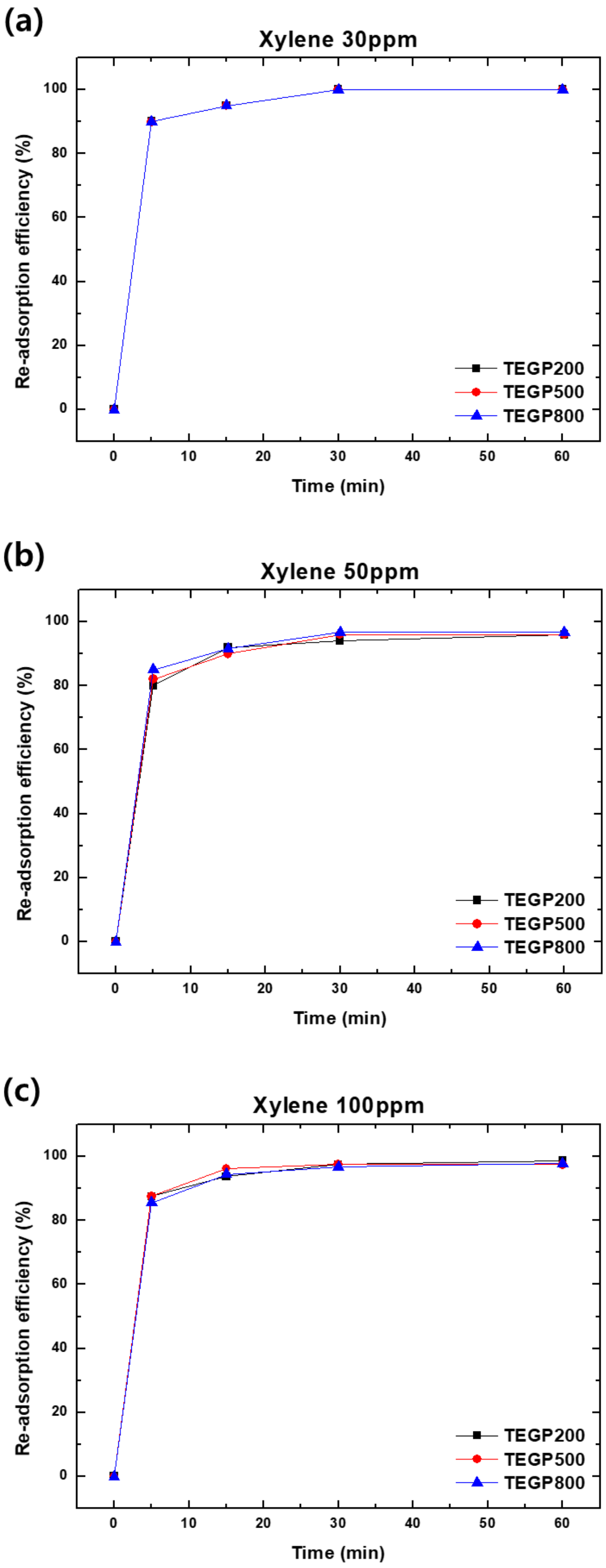

Figure S6. Xylene re-adsorption efficiency according to type of TEGP (a) 30ppm, (b) 50ppm and (c) 100ppm.
